# 2-(4-Chloro­phen­yl)-5-{3,4-dibut­oxy-5-[5-(4-chloro­phen­yl)-1,3,4-oxadiazol-2-yl]thio­phen-2-yl}-1,3,4-oxadiazole

**DOI:** 10.1107/S1600536808035848

**Published:** 2008-11-08

**Authors:** Hai-Lin Li, Hong-Wei Wang, Ran-Zhe Lu, Hai-Bo Wang

**Affiliations:** a College of Science, Nanjing University of Technology, Xinmofan Road No.5 Nanjing, Nanjing 210009, People’s Republic of China; b Nantong Entry–Exit Inspection and Quarantine Bureau, Nantong Jiangsu 226005, People’s Republic of China

## Abstract

In the title compound, C_28_H_26_Cl_2_N_4_O_4_S, the dihedral angles between the two chloro­phenyl rings and the two oxadiazol rings are 10.51 (4)° and 13.55 (3)°, respectively. The thio­phene ring is oriented at dihedral angles of 5.59 (4)°, 8.33 (4)° and 4.41 (4)°, 11.05 (3)°, respectively, with respect to the two oxadiazol and the two chloro­phenyl rings. The intra­molecular C—H⋯O hydrogen bond results in the formation of a five-membered ring. In the crystal structure, π–π contacts between the oxadiazol rings, the chloro­phenyl rings and the chloro­phenyl and oxadiazol rings [centroid–centroid distances = 3.428 (3) Å, 3.750 (3) Å and 3.768 (3) Å, respectively] are present.

## Related literature

For general background, see: Blumstengel *et al.* (1999 ▶); Bugatti *et al.* (2006 ▶); Laurent *et al.* (2005 ▶). For bond-length data, see: Allen *et al.* (1987 ▶).
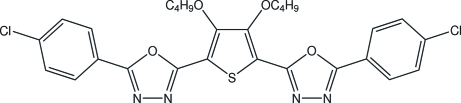


## Experimental

### 

#### Crystal data

C_28_H_26_Cl_2_N_4_O_4_S
*M* *_r_* = 585.49
Monoclinic, 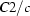

*a* = 19.215 (4) Å
*b* = 22.847 (5) Å
*c* = 14.933 (3) Å
β = 121.25 (3)°
*V* = 5605 (3) Å^3^
*Z* = 8
Mo *K*α radiation
μ = 0.35 mm^−1^
*T* = 294 (2) K
0.30 × 0.10 × 0.10 mm

#### Data collection

Enraf–Nonius CAD-4 diffractometer
Absorption correction: ψ scan (North *et al.*, 1968 ▶) *T*_min_ = 0.903, *T*_max_ = 0.966
10353 measured reflections
5053 independent reflections
2050 reflections with *I* > 2σ(*I*)
*R*_int_ = 0.066
3 standard reflections frequency: 120 min intensity decay: none

#### Refinement

*R*[*F* ^2^ > 2σ(*F* ^2^)] = 0.080
*wR*(*F* ^2^) = 0.198
*S* = 1.01
5053 reflections
298 parameters
H-atom parameters constrained
Δρ_max_ = 0.24 e Å^−3^
Δρ_min_ = −0.46 e Å^−3^

### 

Data collection: *CAD-4 Software* (Enraf–Nonius, 1989 ▶); cell refinement: *CAD-4 Software*; data reduction: *XCAD4* (Harms & Wocadlo, 1995 ▶); program(s) used to solve structure: *SHELXS97* (Sheldrick, 2008 ▶); program(s) used to refine structure: *SHELXL97* (Sheldrick, 2008 ▶); molecular graphics: *SHELXTL* (Sheldrick, 2008 ▶); software used to prepare material for publication: *SHELXTL*.

## Supplementary Material

Crystal structure: contains datablocks I. DOI: 10.1107/S1600536808035848/hk2553sup1.cif

Structure factors: contains datablocks I. DOI: 10.1107/S1600536808035848/hk2553Isup2.hkl

Additional supplementary materials:  crystallographic information; 3D view; checkCIF report

## Figures and Tables

**Table 1:** Hydrogen-bond geometry (Å, °)

*D*—H⋯*A*	*D*—H	H⋯*A*	*D*⋯*A*	*D*—H⋯*A*
C4—H4*B*⋯O1	0.97	2.57	3.203 (7)	123

**Fig. 1.**
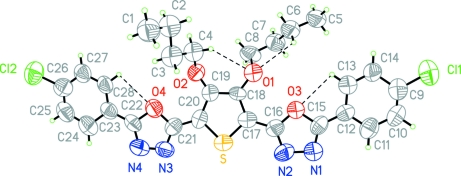
The molecular structure of the title molecule, with the atom-numbering scheme. Displacement ellipsoids are drawn at the 30% probability level.

## supplementary crystallographic information

### Comment

Thiophene derivatives possess electroluminescence (Blumstengel *et al.*, 1999; Bugatti *et al.*,  2006) and biological (Laurent *et al.*, 2005) properties. As part of our studies in this area, we report herein the synthesis and crystal structure of the title compound.

In the title compound (Fig. 1), the bond lengths (Allen *et al.*, 1987) and angles are within normal ranges. Rings A (C9-C14), B (N1/N2/O3/C15/C16), C (S/C17-C20), D (N3/N4/O4/C21/C22) and E (C23-C28) are, of course, planar and the dihedral angles between them are A/B = 7.54 (3)°, A/C = 4.41 (4)°, A/D = 6.88 (4)°, A/E = 10.51 (4)°, B/C = 5.59 (4)°, B/D = 13.55 (3)°, B/E = 16.57 (3)°, C/D = 8.33 (4)°, C/E = 11.05 (3)° and D/E = 3.78 (3)°. The intramolecular C-H···O hydrogen bonds (Table 1) result in the formation of three five- and one six-membered rings F (O3/C12/C13/C15/H13A), G (O1/C6-C8/H6A), H (O4/C22/C23/C28/H28A) and I (O1/O2/C4/C18/C19/H4B). Rings F and H are planar and they are oriented with respect to the adjacent rings at dihedral angles of A/F = 4.18 (4)°, B/F = 8.02 (4)°, D/I = 3.47 (4)° and E/I = 2.05 (4)°. Ring G adopts envelope conformation with C7 atom displaced by 0.570 (3) Å from the plane of the other ring atoms, while ring I has twisted conformation.

In the crystal structure, the π—π contacts between A, D and E rings, Cg3···Cg3^i^, Cg4···Cg4^i^ and Cg5···Cg3^ii^ [symmetry codes: (i) -x, y, -1/2 - z; (ii) -x, -y, -z, where Cg3, Cg4 and Cg5 are the centroids of the rings D (N3/N4/O4/C21/C22), A (C9-C14) and E (C23-C28) may stabilize the structure, with centroid-centroid distances of 3.428 (3) Å, 3.750 (3) Å and 3.768 (3) Å, respectively.

### Experimental

For the preparation of the title compound, 3,4-dibutoxythiophene-2,5-dicarbo- hydrazide (10 mmol) was dissolved in pyridine (30 ml), and then 4-chlorobenzoyl chloride (22 mmol) was added dropwise. The resulting mixture was kept at 345 K for 12 h. After cooling, the mixture was poured into cold water. After filtration and dryness, the colorless solid compound was obtained. The crude compound dissolved in phosphoryl trichloride (30 ml). The mixture was refluxed for 12 h. After cooling, the mixture was poured into smash ice. Then, the title compound was obtained and purified by recrystalization from trichloro- methane (yield; 82.8%, m.p. 451 K). Crystals suitable for X-ray analysis were obtained by slow evaporation of an ethyl acetate solution.

### Refinement

H atoms were positioned geometrically, with C-H = 0.93, 0.97 and 0.96 Å for aromatic, methylene and methyl H, respectively, and constrained to ride on their parent atoms with U_iso_(H) = xU_eq_(C), where x = 1.5 for methyl H and x = 1.2 for all other H atoms.

### Crystal data

**Table:**

C_28_H_26_Cl_2_N_4_O_4_S	*F*_000_ = 2432
*M**_r_* = 585.49	*D*_x_ = 1.388 Mg m^−^^3^
Monoclinic, *C*2/*c*	Melting point: 451K K
Hall symbol: -C 2yc	Mo *K*α radiation λ = 0.71073 Å
*a* = 19.215 (4) Å	Cell parameters from 25 reflections
*b* = 22.847 (5) Å	θ = 9–12º
*c* = 14.933 (3) Å	µ = 0.35 mm^−^^1^
β = 121.25 (3)º	*T* = 294 (2) K
*V* = 5605 (3) Å^3^	Block, yellow
*Z* = 8	0.30 × 0.10 × 0.10 mm

### Data collection

**Table:**

Enraf–Nonius CAD-4 diffractometer	*R*_int_ = 0.066
Radiation source: fine-focus sealed tube	θ_max_ = 25.2º
Monochromator: graphite	θ_min_ = 1.5º
*T* = 294(2) K	*h* = −22→19
ω/2θ scans	*k* = 0→27
Absorption correction: ψ scan(North *et al.*, 1968)	*l* = 0→17
*T*_min_ = 0.903, *T*_max_ = 0.966	3 standard reflections
10353 measured reflections	every 120 min
5053 independent reflections	intensity decay: none
2050 reflections with *I* > 2σ(*I*)	

### Refinement

**Table:**

Refinement on *F*^2^	Secondary atom site location: difference Fourier map
Least-squares matrix: full	Hydrogen site location: inferred from neighbouring sites
*R*[*F*^2^ > 2σ(*F*^2^)] = 0.080	H-atom parameters constrained
*wR*(*F*^2^) = 0.198	*w* = 1/[σ^2^(*F*_o_^2^) + (0.060*P*)^2^ + 7.*P*] where *P* = (*F*_o_^2^ + 2*F*_c_^2^)/3
*S* = 1.01	(Δ/σ)_max_ < 0.001
5053 reflections	Δρ_max_ = 0.24 e Å^−^^3^
298 parameters	Δρ_min_ = −0.46 e Å^−^^3^
Primary atom site location: structure-invariant direct methods	Extinction correction: none

### Special details

**Table:**

Geometry. All e.s.d.'s (except the e.s.d. in the dihedral angle between two l.s. planes) are estimated using the full covariance matrix. The cell e.s.d.'s are taken into account individually in the estimation of e.s.d.'s in distances, angles and torsion angles; correlations between e.s.d.'s in cell parameters are only used when they are defined by crystal symmetry. An approximate (isotropic) treatment of cell e.s.d.'s is used for estimating e.s.d.'s involving l.s. planes.
Refinement. Refinement of *F*^2^ against ALL reflections. The weighted *R*-factor *wR* and goodness of fit *S* are based on *F*^2^, conventional *R*-factors *R* are based on *F*, with *F* set to zero for negative *F*^2^. The threshold expression of *F*^2^ > σ(*F*^2^) is used only for calculating *R*-factors(gt) *etc*. and is not relevant to the choice of reflections for refinement. *R*-factors based on *F*^2^ are statistically about twice as large as those based on *F*, and *R*- factors based on ALL data will be even larger.

### Fractional atomic coordinates and isotropic or equivalent isotropic displacement parameters (Å^2^)

**Table:**

	*x*	*y*	*z*	*U*_iso_*/*U*_eq_	
S	0.42931 (8)	0.23942 (6)	0.30296 (10)	0.0831 (4)	
Cl1	0.55998 (13)	−0.21791 (7)	0.42942 (15)	0.1331 (6)	
Cl2	0.69885 (12)	0.65697 (7)	0.60696 (15)	0.1313 (6)	
O1	0.6213 (2)	0.15456 (15)	0.5028 (3)	0.089	
O2	0.6482 (2)	0.28280 (14)	0.5192 (3)	0.0878 (10)	
O3	0.49888 (19)	0.07239 (14)	0.3740 (2)	0.077	
O4	0.5531 (2)	0.38674 (15)	0.4339 (3)	0.0912 (10)	
N1	0.3730 (3)	0.0474 (2)	0.2575 (3)	0.089	
N2	0.3786 (3)	0.1093 (2)	0.2660 (3)	0.0923 (12)	
N3	0.4340 (3)	0.3757 (2)	0.2900 (4)	0.0953 (13)	
N4	0.4475 (3)	0.4330 (2)	0.3069 (4)	0.0969 (13)	
C1	0.6637 (4)	0.3351 (3)	0.8279 (5)	0.108	
H1B	0.6939	0.3319	0.9028	0.161*	
H1C	0.6102	0.3191	0.8006	0.161*	
H1D	0.6592	0.3756	0.8083	0.161*	
C2	0.7039 (4)	0.3043 (3)	0.7869 (4)	0.108	
H2B	0.7086	0.2630	0.8044	0.129*	
H2C	0.7577	0.3202	0.8115	0.129*	
C3	0.6462 (4)	0.3145 (3)	0.6681 (4)	0.109	
H3B	0.5907	0.3034	0.6451	0.131*	
H3C	0.6473	0.3549	0.6490	0.131*	
C4	0.6818 (3)	0.2761 (3)	0.6269 (4)	0.100	
H4A	0.7398	0.2836	0.6625	0.120*	
H4B	0.6743	0.2360	0.6413	0.120*	
C5	0.7651 (3)	−0.0001 (3)	0.6258 (4)	0.1016 (17)	
H5A	0.7620	−0.0206	0.6798	0.152*	
H5B	0.8207	0.0012	0.6432	0.152*	
H5C	0.7327	−0.0202	0.5603	0.152*	
C6	0.7339 (4)	0.0605 (2)	0.6166 (5)	0.1031 (18)	
H6A	0.6783	0.0585	0.6011	0.124*	
H6B	0.7661	0.0798	0.6838	0.124*	
C7	0.7355 (4)	0.0965 (3)	0.5357 (4)	0.0981 (16)	
H7A	0.7052	0.0757	0.4697	0.118*	
H7B	0.7916	0.0987	0.5530	0.118*	
C8	0.7034 (3)	0.1572 (3)	0.5182 (5)	0.1018 (18)	
H8A	0.7386	0.1815	0.5783	0.122*	
H8B	0.7009	0.1738	0.4569	0.122*	
C9	0.5230 (4)	−0.1491 (2)	0.3946 (5)	0.0945 (16)	
C10	0.4501 (4)	−0.1366 (3)	0.3208 (5)	0.1019 (18)	
H10A	0.4140	−0.1669	0.2841	0.122*	
C11	0.4230 (4)	−0.0771 (3)	0.2937 (5)	0.0971 (16)	
H11A	0.3714	−0.0694	0.2367	0.117*	
C12	0.4728 (3)	−0.0316 (2)	0.3514 (3)	0.0728 (12)	
C13	0.5496 (3)	−0.0449 (2)	0.4331 (4)	0.089	
H13A	0.5834	−0.0144	0.4734	0.106*	
C14	0.5787 (4)	−0.1012 (2)	0.4579 (4)	0.0952 (16)	
H14A	0.6314	−0.1088	0.5125	0.114*	
C15	0.4457 (3)	0.0271 (2)	0.3244 (4)	0.0823 (14)	
C16	0.4529 (3)	0.1223 (2)	0.3318 (4)	0.0801 (13)	
C17	0.4872 (3)	0.1802 (2)	0.3644 (3)	0.0730 (12)	
C18	0.5670 (3)	0.1949 (2)	0.4454 (3)	0.0656 (11)	
C19	0.5800 (3)	0.2557 (2)	0.4558 (4)	0.077	
C20	0.5068 (3)	0.2866 (2)	0.3816 (4)	0.0741 (13)	
C21	0.4949 (3)	0.3481 (2)	0.3638 (4)	0.0786 (13)	
C22	0.5193 (3)	0.4405 (2)	0.3924 (4)	0.0817 (14)	
C23	0.5637 (3)	0.4922 (2)	0.4449 (4)	0.0796 (14)	
C24	0.5285 (4)	0.5466 (3)	0.4006 (5)	0.1096 (19)	
H24A	0.4771	0.5499	0.3407	0.132*	
C25	0.5782 (4)	0.5975 (3)	0.4552 (5)	0.1068 (19)	
H25A	0.5601	0.6344	0.4262	0.128*	
C26	0.6473 (4)	0.5927 (3)	0.5436 (5)	0.0986 (16)	
C27	0.6769 (4)	0.5396 (3)	0.5885 (4)	0.0925 (16)	
H27A	0.7251	0.5370	0.6532	0.111*	
C28	0.6361 (3)	0.4912 (2)	0.5388 (4)	0.0824 (14)	
H28A	0.6578	0.4551	0.5694	0.099*	

### Atomic displacement parameters (Å^2^)

**Table:**

	*U*^11^	*U*^22^	*U*^33^	*U*^12^	*U*^13^	*U*^23^
S	0.0709 (8)	0.0945 (9)	0.0751 (8)	0.0016 (7)	0.0317 (7)	0.0012 (7)
Cl1	0.1665 (17)	0.0968 (11)	0.1486 (15)	0.0058 (11)	0.0905 (14)	0.0078 (10)
Cl2	0.1615 (17)	0.1022 (12)	0.1372 (14)	−0.0175 (11)	0.0825 (13)	−0.0107 (10)
O1	0.082 (3)	0.088 (3)	0.099 (2)	0.0027 (14)	0.0451 (11)	0.0125 (12)
O2	0.082 (2)	0.086 (2)	0.087 (2)	−0.0029 (19)	0.0378 (19)	−0.0123 (18)
N1	0.082 (3)	0.088 (3)	0.092 (2)	0.011 (2)	0.036 (3)	0.013 (2)
N2	0.070 (3)	0.106 (3)	0.095 (3)	−0.014 (2)	0.037 (3)	−0.004 (2)
N3	0.069 (3)	0.091 (3)	0.106 (4)	0.008 (2)	0.032 (3)	0.015 (3)
N4	0.079 (3)	0.105 (4)	0.096 (3)	0.014 (3)	0.037 (3)	0.017 (3)
C1	0.108 (3)	0.112 (3)	0.101 (4)	0.010 (3)	0.051 (4)	0.010 (4)
C2	0.108 (3)	0.103 (3)	0.096 (3)	0.011 (4)	0.046 (4)	0.008 (3)
C3	0.109 (4)	0.102 (3)	0.099 (5)	0.012 (5)	0.047 (5)	0.006 (4)
C4	0.100 (3)	0.088 (4)	0.110 (4)	0.008 (4)	0.052 (4)	0.005 (3)
C5	0.087 (4)	0.104 (4)	0.099 (4)	0.013 (3)	0.039 (3)	0.005 (3)
C6	0.089 (4)	0.097 (4)	0.105 (4)	0.009 (3)	0.038 (3)	−0.005 (3)
C7	0.104 (4)	0.111 (5)	0.080 (4)	0.008 (4)	0.049 (3)	0.003 (3)
C8	0.059 (3)	0.126 (5)	0.120 (4)	0.016 (3)	0.046 (3)	0.021 (4)
C9	0.122 (5)	0.092 (4)	0.083 (4)	−0.006 (4)	0.063 (4)	−0.009 (3)
C10	0.103 (5)	0.099 (5)	0.106 (5)	−0.032 (4)	0.056 (4)	−0.019 (4)
C11	0.097 (4)	0.102 (4)	0.110 (4)	−0.018 (4)	0.066 (4)	−0.012 (4)
C12	0.071 (3)	0.092 (4)	0.059 (3)	−0.007 (3)	0.036 (3)	−0.006 (2)
C13	0.089 (4)	0.091 (4)	0.079 (2)	−0.016 (4)	0.036 (4)	−0.004 (3)
C14	0.100 (4)	0.090 (4)	0.082 (4)	−0.012 (3)	0.038 (3)	0.002 (3)
C15	0.081 (4)	0.089 (4)	0.087 (3)	−0.024 (3)	0.050 (3)	−0.012 (3)
O3	0.077 (3)	0.082 (3)	0.080 (3)	−0.012 (4)	0.040 (3)	−0.012 (3)
C16	0.071 (3)	0.089 (4)	0.079 (3)	−0.004 (3)	0.039 (3)	−0.010 (3)
C17	0.066 (3)	0.096 (3)	0.066 (3)	0.002 (3)	0.040 (3)	0.002 (2)
C18	0.058 (3)	0.080 (3)	0.050 (2)	0.008 (2)	0.021 (2)	−0.011 (2)
C19	0.077 (3)	0.097 (3)	0.074 (2)	−0.016 (2)	0.050 (3)	−0.012 (2)
C20	0.088 (3)	0.073 (3)	0.078 (3)	−0.013 (3)	0.056 (3)	−0.010 (2)
C21	0.087 (4)	0.090 (4)	0.067 (3)	−0.011 (3)	0.045 (3)	−0.013 (3)
O4	0.099 (3)	0.083 (2)	0.090 (2)	0.010 (2)	0.048 (2)	0.0039 (19)
C22	0.089 (4)	0.080 (3)	0.080 (3)	0.026 (3)	0.047 (3)	0.020 (3)
C23	0.082 (4)	0.089 (4)	0.079 (4)	0.020 (3)	0.050 (3)	0.008 (3)
C24	0.111 (5)	0.093 (4)	0.130 (5)	0.018 (4)	0.067 (4)	0.022 (4)
C25	0.132 (6)	0.092 (4)	0.106 (5)	0.034 (4)	0.068 (5)	0.022 (4)
C26	0.088 (4)	0.106 (5)	0.108 (5)	−0.003 (4)	0.055 (4)	0.002 (4)
C27	0.101 (4)	0.096 (4)	0.093 (4)	0.017 (4)	0.059 (3)	−0.002 (3)
C28	0.091 (4)	0.079 (3)	0.084 (4)	0.020 (3)	0.051 (3)	0.001 (3)

### Geometric parameters (Å, °)

**Table:**

S—C17	1.689 (5)	C7—H7B	0.9700
S—C20	1.716 (5)	C8—H8A	0.9700
Cl1—C9	1.693 (6)	C8—H8B	0.9700
Cl2—C26	1.748 (6)	C9—C10	1.287 (8)
O1—C18	1.321 (5)	C9—C14	1.478 (7)
O1—C8	1.472 (5)	C10—C11	1.437 (8)
O2—C19	1.307 (5)	C10—H10A	0.9300
O2—C4	1.397 (6)	C11—C12	1.373 (7)
N1—C15	1.310 (6)	C11—H11A	0.9300
N1—N2	1.419 (6)	C12—C13	1.375 (7)
N2—C16	1.279 (6)	C12—C15	1.419 (7)
N3—C21	1.282 (6)	C13—C14	1.375 (7)
N3—N4	1.333 (6)	C13—H13A	0.9300
N4—C22	1.318 (6)	C14—H14A	0.9300
C1—C2	1.400 (7)	C15—O3	1.369 (5)
C1—H1B	0.9600	O3—C16	1.378 (6)
C1—H1C	0.9600	C16—C17	1.445 (7)
C1—H1D	0.9600	C17—C18	1.415 (6)
C2—C3	1.546 (8)	C18—C19	1.408 (6)
C2—H2B	0.9700	C19—C20	1.445 (7)
C2—H2C	0.9700	C20—C21	1.426 (7)
C3—C4	1.432 (7)	C21—O4	1.381 (6)
C3—H3B	0.9700	O4—C22	1.377 (5)
C3—H3C	0.9700	C22—C23	1.429 (7)
C4—H4A	0.9700	C23—C28	1.370 (7)
C4—H4B	0.9700	C23—C24	1.407 (7)
C5—C6	1.486 (7)	C24—C25	1.456 (9)
C5—H5A	0.9600	C24—H24A	0.9300
C5—H5B	0.9600	C25—C26	1.304 (8)
C5—H5C	0.9600	C25—H25A	0.9300
C6—C7	1.475 (7)	C26—C27	1.360 (8)
C6—H6A	0.9700	C27—C28	1.337 (7)
C6—H6B	0.9700	C27—H27A	0.9300
C7—C8	1.484 (7)	C28—H28A	0.9300
C7—H7A	0.9700		
			
C17—S—C20	92.2 (2)	C9—C10—H10A	119.2
C18—O1—C8	119.7 (4)	C11—C10—H10A	119.2
C19—O2—C4	118.2 (4)	C12—C11—C10	120.6 (6)
C15—N1—N2	106.5 (4)	C12—C11—H11A	119.7
C16—N2—N1	107.6 (4)	C10—C11—H11A	119.7
C21—N3—N4	108.6 (5)	C11—C12—C13	117.8 (5)
C22—N4—N3	108.2 (4)	C11—C12—C15	120.3 (5)
C2—C1—H1B	109.5	C13—C12—C15	121.8 (5)
C2—C1—H1C	109.5	C12—C13—C14	123.1 (5)
H1B—C1—H1C	109.5	C12—C13—H13A	118.5
C2—C1—H1D	109.5	C14—C13—H13A	118.5
H1B—C1—H1D	109.5	C13—C14—C9	117.5 (5)
H1C—C1—H1D	109.5	C13—C14—H14A	121.3
C1—C2—C3	101.3 (5)	C9—C14—H14A	121.3
C1—C2—H2B	111.5	N1—C15—O3	110.2 (5)
C3—C2—H2B	111.5	N1—C15—C12	129.8 (5)
C1—C2—H2C	111.5	O3—C15—C12	120.0 (5)
C3—C2—H2C	111.5	C15—O3—C16	104.8 (4)
H2B—C2—H2C	109.3	N2—C16—O3	110.8 (5)
C4—C3—C2	100.5 (5)	N2—C16—C17	127.1 (5)
C4—C3—H3B	111.7	O3—C16—C17	122.1 (4)
C2—C3—H3B	111.7	C18—C17—C16	127.4 (4)
C4—C3—H3C	111.7	C18—C17—S	113.0 (4)
C2—C3—H3C	111.7	C16—C17—S	119.6 (4)
H3B—C3—H3C	109.4	O1—C18—C19	125.6 (4)
O2—C4—C3	113.0 (5)	O1—C18—C17	122.1 (4)
O2—C4—H4A	109.0	C19—C18—C17	112.4 (4)
C3—C4—H4A	109.0	O2—C19—C18	127.0 (4)
O2—C4—H4B	109.0	O2—C19—C20	122.4 (4)
C3—C4—H4B	109.0	C18—C19—C20	110.5 (4)
H4A—C4—H4B	107.8	C21—C20—C19	128.4 (5)
C6—C5—H5A	109.5	C21—C20—S	119.6 (4)
C6—C5—H5B	109.5	C19—C20—S	111.8 (4)
H5A—C5—H5B	109.5	N3—C21—O4	110.8 (5)
C6—C5—H5C	109.5	N3—C21—C20	129.1 (5)
H5A—C5—H5C	109.5	O4—C21—C20	120.0 (5)
H5B—C5—H5C	109.5	C22—O4—C21	102.8 (4)
C7—C6—C5	114.6 (5)	N4—C22—O4	109.4 (5)
C7—C6—H6A	108.6	N4—C22—C23	131.7 (5)
C5—C6—H6A	108.6	O4—C22—C23	118.8 (5)
C7—C6—H6B	108.6	C28—C23—C24	118.7 (5)
C5—C6—H6B	108.6	C28—C23—C22	123.2 (5)
H6A—C6—H6B	107.6	C24—C23—C22	117.9 (5)
C6—C7—C8	118.8 (5)	C23—C24—C25	115.2 (6)
C6—C7—H7A	107.6	C23—C24—H24A	122.4
C8—C7—H7A	107.6	C25—C24—H24A	122.4
C6—C7—H7B	107.6	C26—C25—C24	121.9 (6)
C8—C7—H7B	107.6	C26—C25—H25A	119.0
H7A—C7—H7B	107.0	C24—C25—H25A	119.0
O1—C8—C7	107.7 (5)	C25—C26—C27	121.3 (6)
O1—C8—H8A	110.2	C25—C26—Cl2	118.1 (5)
C7—C8—H8A	110.2	C27—C26—Cl2	120.5 (5)
O1—C8—H8B	110.2	C28—C27—C26	119.3 (6)
C7—C8—H8B	110.2	C28—C27—H27A	120.4
H8A—C8—H8B	108.5	C26—C27—H27A	120.4
C10—C9—C14	119.3 (6)	C27—C28—C23	123.2 (5)
C10—C9—Cl1	124.4 (5)	C27—C28—H28A	118.4
C14—C9—Cl1	116.3 (5)	C23—C28—H28A	118.4
C9—C10—C11	121.6 (6)		
			
C15—N1—N2—C16	3.0 (5)	C16—C17—C18—C19	179.8 (4)
C21—N3—N4—C22	1.1 (6)	S—C17—C18—C19	−0.4 (5)
C1—C2—C3—C4	−172.2 (5)	C4—O2—C19—C18	−63.3 (6)
C19—O2—C4—C3	−82.5 (6)	C4—O2—C19—C20	119.5 (5)
C2—C3—C4—O2	−171.2 (5)	O1—C18—C19—O2	3.8 (8)
C5—C6—C7—C8	178.2 (5)	C17—C18—C19—O2	−176.0 (4)
C18—O1—C8—C7	−152.1 (4)	O1—C18—C19—C20	−178.7 (4)
C6—C7—C8—O1	−51.8 (7)	C17—C18—C19—C20	1.5 (5)
C14—C9—C10—C11	4.3 (8)	O2—C19—C20—C21	0.1 (8)
Cl1—C9—C10—C11	−178.7 (4)	C18—C19—C20—C21	−177.5 (4)
C9—C10—C11—C12	−4.8 (9)	O2—C19—C20—S	175.7 (3)
C10—C11—C12—C13	1.9 (7)	C18—C19—C20—S	−1.9 (5)
C10—C11—C12—C15	−179.9 (5)	C17—S—C20—C21	177.5 (4)
C11—C12—C13—C14	1.2 (7)	C17—S—C20—C19	1.4 (3)
C15—C12—C13—C14	−176.9 (5)	N4—N3—C21—O4	−0.6 (6)
C12—C13—C14—C9	−1.7 (8)	N4—N3—C21—C20	177.3 (5)
C10—C9—C14—C13	−1.1 (8)	C19—C20—C21—N3	170.1 (5)
Cl1—C9—C14—C13	−178.4 (4)	S—C20—C21—N3	−5.2 (7)
N2—N1—C15—O3	−1.7 (5)	C19—C20—C21—O4	−12.3 (7)
N2—N1—C15—C12	176.4 (5)	S—C20—C21—O4	172.4 (3)
C11—C12—C15—N1	10.1 (8)	N3—C21—O4—C22	−0.2 (5)
C13—C12—C15—N1	−171.9 (5)	C20—C21—O4—C22	−178.2 (4)
C11—C12—C15—O3	−172.0 (4)	N3—N4—C22—O4	−1.2 (6)
C13—C12—C15—O3	6.0 (7)	N3—N4—C22—C23	179.2 (5)
N1—C15—O3—C16	−0.1 (5)	C21—O4—C22—N4	0.9 (5)
C12—C15—O3—C16	−178.4 (4)	C21—O4—C22—C23	−179.5 (4)
N1—N2—C16—O3	−3.2 (5)	N4—C22—C23—C28	174.8 (5)
N1—N2—C16—C17	179.4 (4)	O4—C22—C23—C28	−4.7 (7)
C15—O3—C16—N2	2.2 (5)	N4—C22—C23—C24	−1.1 (8)
C15—O3—C16—C17	179.7 (4)	O4—C22—C23—C24	179.4 (4)
N2—C16—C17—C18	172.2 (5)	C28—C23—C24—C25	6.4 (7)
O3—C16—C17—C18	−5.0 (7)	C22—C23—C24—C25	−177.5 (5)
N2—C16—C17—S	−7.5 (7)	C23—C24—C25—C26	−5.5 (9)
O3—C16—C17—S	175.3 (3)	C24—C25—C26—C27	0.9 (9)
C20—S—C17—C18	−0.6 (3)	C24—C25—C26—Cl2	−175.8 (5)
C20—S—C17—C16	179.2 (4)	C25—C26—C27—C28	3.0 (9)
C8—O1—C18—C19	−49.3 (6)	Cl2—C26—C27—C28	179.6 (4)
C8—O1—C18—C17	130.5 (5)	C26—C27—C28—C23	−1.9 (8)
C16—C17—C18—O1	0.0 (7)	C24—C23—C28—C27	−3.1 (8)
S—C17—C18—O1	179.7 (3)	C22—C23—C28—C27	−179.0 (5)

### Hydrogen-bond geometry (Å, °)

**Table:**

*D*—H···*A*	*D*—H	H···*A*	*D*···*A*	*D*—H···*A*
C4—H4B···O1	0.97	2.57	3.203 (7)	123
